# Inter-individual variability in the foraging behaviour of traplining bumblebees

**DOI:** 10.1038/s41598-017-04919-8

**Published:** 2017-07-04

**Authors:** Simon Klein, Cristian Pasquaretta, Andrew B. Barron, Jean-Marc Devaud, Mathieu Lihoreau

**Affiliations:** 1 Research Center on Animal Cognition, Center for Integrative Biology, National Center for Scientific Research (CNRS), University of Toulouse (UPS), Toulouse, France; 2 Department of Biological Sciences, Macquarie University, Sydney, NSW Australia

## Abstract

Workers of social insects, such as bees, ants and wasps, show some degree of inter-individual variability in decision-making, learning and memory. Whether these natural cognitive differences translate into distinct adaptive behavioural strategies is virtually unknown. Here we examined variability in the movement patterns of bumblebee foragers establishing routes between artificial flowers. We recorded all flower visitation sequences performed by 29 bees tested for 20 consecutive foraging bouts in three experimental arrays, each characterised by a unique spatial configuration of artificial flowers and three-dimensional landmarks. All bees started to develop efficient routes as they accumulated foraging experience in each array, and showed consistent inter-individual differences in their levels of route fidelity and foraging performance, as measured by travel speed and the frequency of revisits to flowers. While the tendency of bees to repeat the same route was influenced by their colony origin, foraging performance was correlated to body size. The largest foragers travelled faster and made less revisits to empty flowers. We discuss the possible adaptive value of such inter-individual variability within the forager caste for optimisation of colony-level foraging performances in social pollinators.

## Introduction

In recent years, behavioural ecologists have become increasingly interested by the fact that animals often exhibit consistent behavioural traits that vary between individuals from the same group, population or species, irrespective of time or context^1–3^. Inter-individual behavioural variability has been described in a wide range of taxa, from invertebrates (nematodes^4^, cnidarians^5^, molluscs^6^, insects^7, 8^) to mammals^9^, including humans^10^. The existence of such individualistic behavioural traits may have different adaptive values depending on the ecology of the species^11–13^.

Social insects, such as ants, some bees and wasps, show extreme cases of inter-individual behavioural variability^14^. In these animals, division of labour typically implies that specific individuals reproduce (the queens and the males), whereas others work to support their reproductive outputs (the workers)^15^. Among the workers different individuals specialise on different roles. Some take care of the brood (the nurses), while others defend the colony entrance (the guards and the soldiers) or collect food (the foragers). These behavioural specialists exhibit specific behavioural repertoires that can be associated with differences in morphology (e.g. bumblebees^16^), age (e.g. honey bees^17^), physiology and genetics (e.g. honey bees^18, 19^), or experience (e.g. ants^20^), together defining the caste phenotype. Growing evidence indicates that some level of behavioural variability also exists between individuals of the same caste^21–23^. For instance in bumblebees, foragers show consistent inter-individual differences in decision speed and accuracy in flower discrimination tasks^24, 25^. When having to choose between a rewarding flower and an empty flower in a laboratory decision chamber, some foragers always make slow but accurate decisions, while others are consistently fast and inaccurate^24^. Foragers also show inter-individual variability in learning performance^22, 26^ and colonies containing foragers with high visual learning speeds have a higher foraging efficiency^27^. These differences are independent of body size or any other measurable morphological attributes^27^.

Whether such cognitive variability translates into distinct foraging strategies in the more complex and ecologically relevant task of exploiting patchily distributed floral resources remains virtually unexplored. In nature, bees often develop stable foraging routes (sometimes called traplines in analogy to trappers checking their traps along fixed routes^28^) to exploit multiple feeding locations from their central nest^29, 30^. Manipulative experiments on bumblebees^31, 32^ and honey bees^33^ foraging for sucrose solution in simple arrays of artificial flowers (equivalent to natural flower patches) show how foragers often find the shortest possible route to visit all flowers once and return to the nest using an iterative improvement strategy based on learning and memory that is different from just linking nearest neighbour locations^31, 34^.

Thus far empirical research on trapline foraging has been aimed at describing this behaviour at the species level, using relatively small sample sizes (four to seven individuals per experiment), without characterising variation among individuals^31–33, 35–38^. In principle however, some level of variation in the foraging behaviour of the workers of a colony could improve the colony foraging efficiency^39^. Regular trapliners that accurately follow the same route across multiple hours or days may perform better in stable environments when resources are highly predictable, while irregular trapliners that sample new locations at each foraging bout may be advantaged in more variable environments. Consequently, colonies containing foragers of different behavioural profiles may differ in performance in similar environmental conditions. Understanding how natural behavioural variability affects the foraging performances of colonies may help evaluate the adaptability of bees in the face of environmental changes, such as natural climatic events, human-induced habitat degradations or the introduction of predators and parasites^40^. Ultimately, this approach may also help refine predictions of current pollination models based on bee movement patterns^34, 38, 39, 41, 42^.

Here we explored the level of inter-individual variability in the foraging behaviour of bumblebees (*Bombus terrestris*) by comparing the movement patterns of foragers from two colonies collecting sucrose solution in three different arrays of artificial flowers and landmarks in a controlled flight room.

## Results

**Figure 1:**
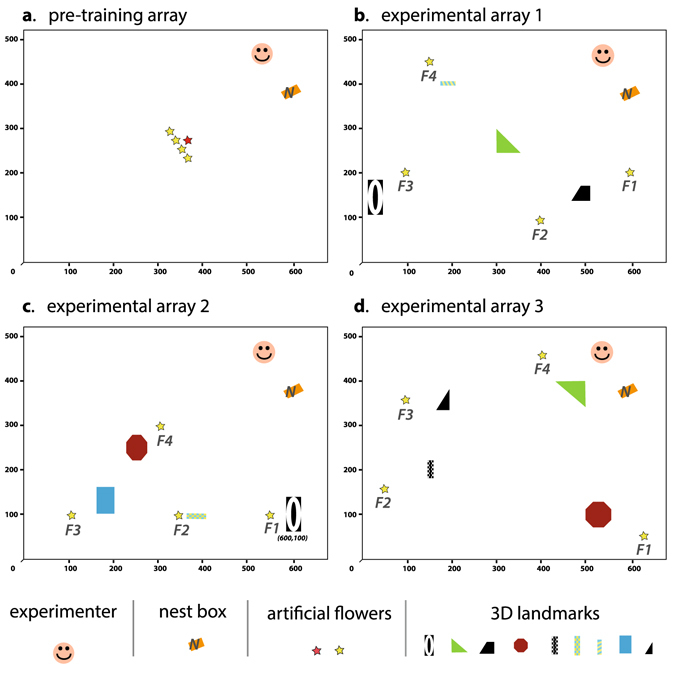
Experimental arrays of flowers and landmarks. (**a**) Pre-training array. Bees were allowed to forage on a pre-training flower (red star) in a landmark-free environment for one hour. A selected bee was then observed foraging on four training flowers (yellow stars) during five foraging bouts to estimate its nectar crop capacity. (**b**–**d**) show the first, second and third experimental arrays used for testing. Each array was characterised by a unique combination of four training flowers (F1-F4) and three to four landmarks (coloured shapes). Detailed descriptions of the artificial flowers and the 3D landmarks are given in Figs S1 and S2. X- and Y-axis graduations represent the distance to the origin (down left corner) in cm.

We tested 29 bees from two colonies (N = 15 from colony 1, N = 14 from colony 2). Each bee was successively observed for 20 consecutive foraging bouts (flower visits followed by returns to the colony nest box) in three experimental arrays each characterised by four flower locations and four different landmarks (Figs 1, S1 and S2). The experimental arrays were chosen in order to maximise the level of dissimilarity between them while keeping a simple design. Bees were tested successively following the same order of arrays presentation. At every foraging bout, each flower contained a volume of sucrose solution equivalent to one quarter of the bee’s nectar crop (stomach) capacity so that the task for the bee was to visit the four flowers to fill its crop to capacity and then return to the nest.

### Bees developed routes in the three experimental arrays

**Figure 2:**
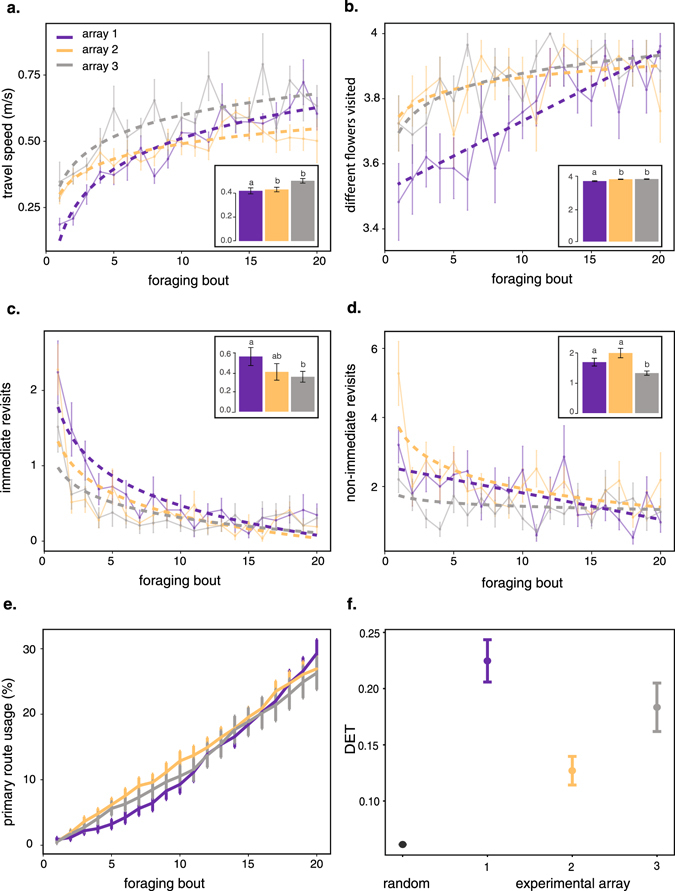
Average behavioural measures in the three experimental arrays (array 1: purple, array 2: orange, array 3: grey, see details of flower and landmark configurations in Fig. 1). (**a**) Travel speed per foraging bout (flight duration divided by the Euclidian distance between all successively visited flowers). (**b**) Number of different flower visited per foraging bout. (**c**) Number of immediate revisits to flowers per foraging bout (when the bee visited the same flower twice in a row). (**d**) Number of non-immediate revisits per foraging bout (when the bee revisited a flower after having visited one or more different flower locations). (**e**) Cumulative frequency of primary route usage per foraging bout. (**a**–**e**) plain lines show means ± SE (N = 29 bees), dashed lines show regression models (see details in Table 1 and Supplementary Table S1). (**f**) Comparison between simulated random determinism index (DETs, N = 1000 simulations) and observed DETs (N = 29 bees) in each experimental array (mean ± SE). (**a**–**d**) Bar plots show means ± SE for each array of flowers. Tukey post-hoc analysis: different letters above bars represent significant differences between arrays (see details in Supplementary Table S2).

We first considered the overall foraging behaviour of bees in all three experimental arrays. On average bees increased by 154.5 ± 48.3% (mean ± SE) their travel speed (flight duration divided by the Euclidian distance between all successively visited flowers) between the first and the last foraging bout in the same array (Fig. 2A, Table 1). Although we used an indirect measure of travel speed, there is clear evidence that bumblebees rapidly develop straight flight trajectories to join known flower locations with training^38, 43^. As they gained experience in an array, bees also increased by 6.3 ± 3.8% (mean ± SE) the average number of different flower locations they visited per bout (Fig. 2B, Table 1), decreased by 85.3 ± 3.5% (mean ± SE) the average number of immediate revisits to flowers (two successive visits to the same flower; Fig. 2C, Table 1), and decreased by 58.0 ± 8.0% (mean ± SE) the average number of non-immediate revisits (two non-successive visits to the same flower; Fig. 2D, Table 1).

**Table 1:** Regression coefficients of average behavioural measures for the three experimental arrays.

	Type of regression	Estimate (SE)	t	P
Travel speed				
**Array 1**	**logarithmic**	**0**.**16 (0**.**01)**	**11**.**04**	**<0**.**001**
**Array 2**	**logarithmic**	**0**.**09 (0**.**02)**	**4**.**35**	**<0**.**001**
**Array 3**	**logarithmic**	**0**.**64 (0**.**11)**	**−1**.**23**	**<0**.**001**
Different flowers visited				
**Array 1**	**linear**	**0**.**02 (0**.**003)**	**7**.**80**	**<0**.**001**
**Array 2**	**logarithmic**	**0**.**05 (0**.**02)**	**2**.**71**	**0**.**014**
**Array 3**	**logarithmic**	**0**.**08 (0**.**02)**	**4**.**57**	**<0**.**001**
Immediate revisits to flowers				
**Array 1**	**logarithmic**	**−0**.**57 (0**.**06)**	**−9**.**33**	**<0**.**001**
**Array 2**	**logarithmic**	**−0**.**43 (0**.**09)**	**−4**.**73**	**<0**.**001**
**Array 3**	**logarithmic**	**−0**.**29 (0**.**06)**	**−5**.**13**	**<0**.**001**
Non-immediate revisits to flowers				
**Array 1**	**linear**	**−0**.**08 (0**.**02)**	**−3**.**42**	**0**.**003**
**Array 2**	**logarithmic**	**−0**.**77 (0**.**18)**	**−4**.**34**	**<0**.**001**
**Array 3**	**logarithmic**	**−0.14 (0.11)**	**−1.25**	**0. 228**

Significant effects are highlighted in bold.

We estimated the tendency of bees to follow regular routes over repeated foraging bouts by calculating the frequency of use of a primary route (highest proportion of foraging bouts in which the same four-flowers visitations sequence — excluding revisits to flowers — was used by a bee)^36^. Each bee established a primary route that it used on average in 27.5 ± 2.2% (mean ± SE) of all its foraging bouts for a given array (Fig. 2E). This proportion of primary route usage was similar in the three experimental arrays (Kruskall-Wallis test: χ^2^ = 1.47, P = 0.478). We calculated the level of similarity between the 20 complete flower visitation sequences for each bee in each experimental array using a determinism index (DET). This index is derived from recurrence quantification analyses that reflect the amount of repeated sequences in a dataset^44^. DET varies between 0 (the bee never repeats the same flower visitations sequence) and 1 (the bee always repeats the same flower visitations sequence). For all three arrays, observed DETs were consistently higher than theoretical DETs calculated on simulated random flower visitations sequences (Fig. 2F; post-hoc Tukey test, array 1: β = 0.16 ± 0.01, t = 30.41, P < 0.001; array 2: β = 0.07 ± 0.01, t = 12.22, P < 0.001; array 3: β = 0.12 ± 0.01, t = 22.72, P < 0.001). This indicates that bee movement patterns were more repeatable than expected by chance. Thus, overall bees increased their foraging efficiency and began to develop traplines as they accumulated foraging experience in each array, irrespective of the spatial distribution of flowers and the nature and arrangement of three-dimensional landmarks.

Nonetheless, some behavioural differences were observed for all bees between the three arrays. For instance, in array 1 bees tended to travel slower (Fig. 2A, Supplementary Table S2), visited fewer flowers (Fig. 2B, Supplementary Table S2) and tended to perform more immediate revisits (Fig. 2C, Supplementary Table S2), while they performed fewer non-immediate revisits in array 3 (Fig. 2D, Supplementary Table S2). This suggests that bees continuously improved their foraging performance throughout the experiment, as they accumulated experience from the first to the third array. However we cannot exclude that these changes of foraging performance also reflect differences in the degree of navigational challenge offered by each array and their sequences of presentation. For instance bees appeared to have lower DETs in array 2 (least-squares means post-hoc test: array 2 *vs*. array 1: P < 0.001; array 1 *vs*. array 3: P = 0.072; array 2 *vs*. array 3: P = 0.031). In this case flower 2 may have been particularly difficult to locate as it was hidden behind a tall landmark.

### Bees showed strong variability in route fidelity and foraging performance

**Figure 3:**
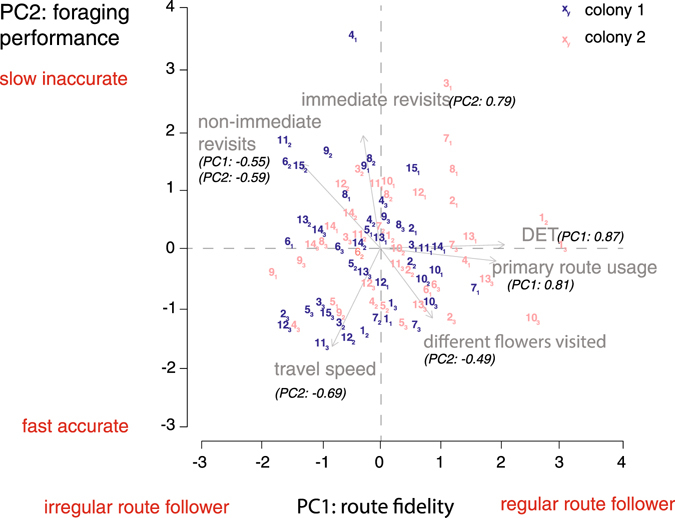
Correlations between the two first components (PCs) of the principal component analysis (PCA). Grey arrows represent the six behavioural measures on PC1 (route fidelity) and PC2 (foraging performance). PC loadings are in brackets. Only loadings >|0.4| were retained (see Supplementary Table S3 for the complete PCA loadings). Each data point represents the PC1 and PC2 scores of a given bee in each experimental array. The PCs define a continuum between four behavioural extremes: fast accurate and regular route followers, fast accurate and irregular route followers, slow inaccurate and regular route followers, slow inaccurate and irregular route followers. Blue: colony 1 (N = 15 bees, 45 data points), red: colony 2 (N = 14 bees, 42 data points). Numbers refer to individual bees (same number code as in Figs 4 and 5). Subscripts refer to experimental arrays (1–3).

Having described the average foraging behaviour of bees in the three arrays, we next explored the level of inter-individual variability among the different foragers. We ran a principal component analysis (PCA) based on the mean for each individuals per array for the six behavioural measures described above: (1) travel speed per foraging bout (flight duration divided by the Euclidian distance between all successively visited flowers); (2) number of different flowers visited per foraging bout; (3) number of immediate revisits to flowers per foraging bout (when the bee visited the same flower twice in a row); (4) number of non-immediate revisits per foraging bout (when the bee revisited a flower after having visited one or more different flowers); (5) cumulative frequency of primary route usage per foraging bout; (6) determinism index (DET, level of similarity between the 20 flower visitation sequences) for each experimental array; Figs 3 and S3). We retained two PCs using the Kaiser-Guttman criterion (Supplementary Fig. S4).

PC1 and PC2 were not correlated with each other (Spearman’s correlation test: ρ = 0.01, S = 108460, P = 0.915). PC1 explained 54% of the proportion and PC2 46%. PC1 was positively associated with the frequency of use of a primary route and the DET, but negatively associated with the number of non-immediate revisits to flowers (Fig. 3, Supplementary Table S3). We interpreted PC1 as a “route fidelity” variable. Accordingly individuals with a high PC1 score were regular route-followers characterised by highly repeatable flower visitation sequences and occasional non-immediate revisits to flowers. PC2 was positively associated with the number of immediate and non-immediate revisits to flowers, and negatively associated with travel speed and the number of different flowers visited (Fig. 3, Supplementary Table S3). We interpreted PC2 as a “foraging performance” variable. Individuals with a high PC2 score were slow and inaccurate foragers, characterised by slow movements between flowers and frequent revisits to empty flowers. Variance along PC1 and PC2 defined a continuum between four behavioural extremes (Fig. 3): fast accurate and regular route followers (high PC1/low PC2 scores), fast accurate and irregular route-followers (low PC1/low PC2 scores), slow inaccurate and regular route-followers (high PC1/high PC2 scores), and slow inaccurate and irregular route-followers (low PC1/high PC2 scores). While foragers of colony 2 were uniformly distributed across the entire PC space, 50% of the foragers of colony 1 were nested within the area defined by high PC1 and low PC2 scores (slow inaccurate and irregular route-followers; Fig. 3).

### Variability was expressed both at the inter- and intra-individual levels

We next explored the effects of inter- and intra-individual variability on PC1 and PC2, using linear mixed effect models (LMMs) with individual identity nested within colony identity as random effects and both intercept (inter-individual variability) and random slope (intra-individual variability) structures.

**Figure 4:**
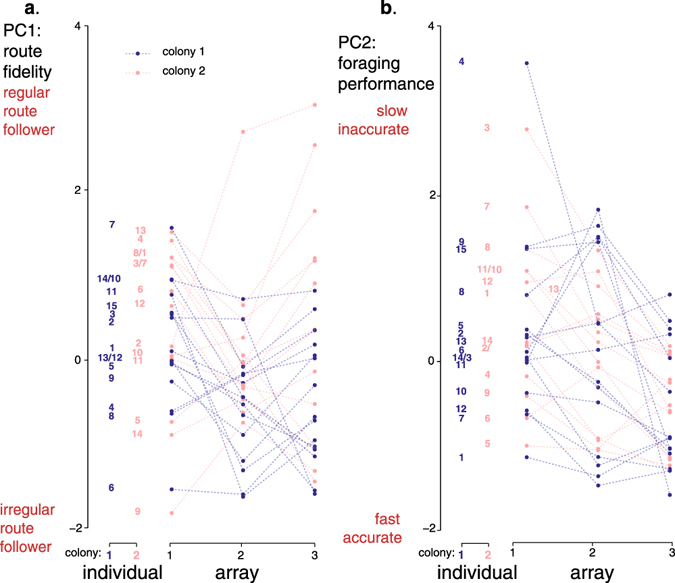
Intra- and inter-individual behavioural variance across experimental arrays. (**a**) Route fidelity (PC1). (**b**) Foraging performance (PC2). Data points connected by a dashed-line represent the scores of the same individual over the three arrays. Blue: colony 1 (N = 15 bees), red: colony 2 (N = 14 bees). Numbers refer to individual bees (the same number code was used in Figs 3 and 5).

Variability in PC1 was significantly explained by inter-individual differences (Table 2A; 27% of variance explained), meaning that bees showed consistent differences in their average level of route fidelity across arrays. Bees also differed in their level of intra-individual variability (Table 2B; 11% of variance explained) so that some individuals consistently increased their route fidelity in each array while others did not. Variability in PC1 was also explained by differences between colonies (Table 2A; 38% of variance explained). Overall bees from colony 2 were more regular at following a route than bees from colony 1, irrespective of the experimental array (Fig. 4A).

**Table 2:** Log-likelihood Ratio tests to estimate inter- and intra-individual variability on the two principal components (PCs) of the principal component analysis (PCA).

	df	AIC	Loglik	L.Ratio	P
(**a**)					
*Random intercept model PC1*					
LM	5	262.67	−126.34		
LME_1|colony	6	228.64	−108.32	7.08	0.008
**LME_1|colony/ID**	**7**	**254**.**48**	**−120**.**24**	**5**.**11**	**0**.**024**
*Random intercept model PC2*					
LM	5	239.54	−114.77		
LME_1|colony	6	237.84	−112.92	3.70	0.054
**LME_1|colony/ID**	**7**	**225**.**13**	**−105**.**57**	**14**.**72**	**<0**.**001**
(**b**)					
*Random slope model PC1*					
LME_1|colony/ID	7	242.57	−114.29		
**LME_0+array|colony/ID**	**6**	**235**.**93**	**−111**.**96**	**4**.**64**	**0**.**031**
*Random slope model PC2*					
**LME_1|colony/ID**	**7**	**201**.**92**	**−98**.**46**		
LME_0+array|colony/ID	6	227.93	−107.92	19.00	<0.001

(**a**) To study inter-individual variability we compared a linear model (LM) built using each PC as a response variable and age, body size and experimental array as fixed variables with two mixed effect models (LMEs) using colony or individual nested in colony as random effects. (**b**) To study intra-individual variability we compared the random intercept model (LME_1|colony/ID) previously built using each PC with a random intercept and slope model (LME_0+array|colony/ID). Degree of freedom (df), Akaike Information Criterion (AIC), Log-likelihood values (Loglik) and Log-likelihood ratio test (L.Ratio) are presented with the corresponding p-values. Significant effects are highlighted in bold.

Variability in PC2 was significantly explained by inter-individual differences (Table 2A; 46% of variance explained). Therefore bees showed consistent differences in their average level of route performance across arrays. Bees did not present intra-individual variability in their response to the different arrays (Table 2B; 5% of variance explained), meaning that all bees tended to increase their foraging performance as they gained experience in a given array. Colony origin had no effect on PC2 (Table 2A; 26% of variance explained).

### Body size differences partly explain inter-individual variability in foraging performances

**Figure 5:**
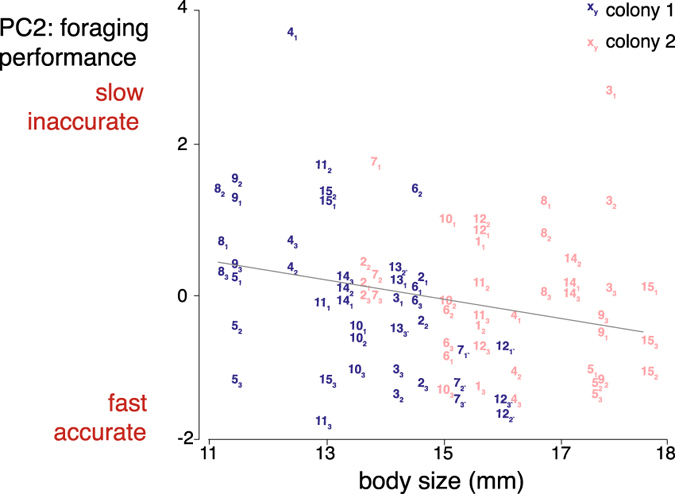
Inter-individual variance in foraging performance (PC2) is partly explained by body size (length from top of head to end of abdomen). Each data point represents the average score of an individual in an experimental array (three values per individual). Blue: colony 1 (N = 15 bees), red: colony 2 (N = 13 bees). Numbers refer to individual bees (the same number code was used in Figs 3 and 4). Subscripts refer to experimental arrays (1–3). Marginal R^2^ = 0.12, conditional R^2^ = 0.44.

We used LMMs to examine whether experimental factors (spatial configuration of flowers and landmarks) or biological characteristics of bees (body size and age) explained both PCs (Table 3). PC1 was neither explained by experimental arrays, body size or age (Table 3). By contrast PC2 was negatively correlated with body size, so that larger foragers tended to travel faster and make fewer revisits to flowers than smaller foragers (Fig. 5). We also found a significant influence of the experimental arrays on PC2 (Table 3), indicating that bees similarly increased their foraging performance as they moved from array 1 to array 2 and array 3 (Fig. 4B). This gradual improvement of foraging performances supports the hypothesis of a continuous learning process throughout the experiment.

**Table 3:** Linear mixed models (LMMs).

	Estimate (SE)	df	t	P
*Route fidelity (PC1)*				
Body size	−0.12 (0.09)	24	−1.38	0.190
Age	−0.01 (0.02)	24	−0.37	0.709
Array	−0.18 (0.11)	55	−1.23	0.116
*Foraging performance (PC2)*				
**Body size**	**−0**.**21 (0**.**09)**	**24**	**−2**.**36**	**0**.**03**
Age	−0.01 (0.02)	24	−0.53	0.60

LMMs were run on the two principal components (PCs) of the principal component analysis (PCA), using individual identity nested within colony identity as random variables and age, body size and experimental array as fixed variables. Significant effects are highlighted in bold.

## Discussion

Understanding inter-individual behavioural variability in complex societies, such as colonies of social insects, may offer unique insights into how and why relatively high levels of inter-individual behavioural variability are observed in animal groups and populations^22, 45^. Here we compared the movement patterns of all foragers from two bumblebee colonies exploiting arrays of stable feeder locations, and report consistent inter-individual differences in their spatial foraging behaviour. Rather than defining distinct behavioural profiles of foragers, this natural variability follows a continuum along two behavioural dimensions. Some bees were always more faithful to a route and/or faster and more accurate in their spatial foraging decisions than others.

Bees showed consistent inter-individual variability in their tendency to follow stable routes between flowers. This variability was neither explained by the characteristics of our experimental arrays of flowers and landmarks, nor the body size or the age of bees. Interestingly, degrees of route fidelity differed between our two colonies, meaning that foragers from one colony were more regular in following a route than those from the other colony. These results are not due to differences in the average body size or age between the foragers of each colony. Behavioural variability between individuals of different groups or colonies is a widespread phenomenon in social animals^45^, including insects^21, 46–48^. Inter-colonial behavioural variability has been reported previously in bees, (e.g. aggression in honey bees^49^ or for both vision- and olfaction-related cognitive tasks in bumblebees^27^) and suggested to be correlated with the foraging success of colonies^26, 27^. In bumblebees, high genetic relatedness between colony members, due to female monandry (single mating) and haplo-diploidy (haploid males, diploid females), may favour strong inter-colony variability^26, 50^. Other non-genetic factors may also contribute to phenotypic variability between colonies, such as changes in the pre-imaginal environment. For instance variation in nest temperature^51^ and nutrition^52^ during the larval stage can lead to differences in olfactory learning in adult honey bees. Further studies using more colonies with known genetic relatedness are needed to test the existence of a genetically determined inter-colony variability for traplining.

In the present spatial task, bees also showed some level of inter-individual variability in their ability to make fast and accurate spatial decisions, so that fast travelling bees made fewer revisits to empty flowers. This result is consistent with the observation that goal-directed flights in experienced bees, for instance between the nest and familiar flowers, are faster than exploration flights, in which naïve bees scan the environment to search for flowers and acquire spatial memories^38, 43^. Thus potentially bees showed inter-individual variability in their tendency to make exploitation and exploration flights. Interestingly, differences in foraging performance among bumblebee foragers were partly explained by differences in their body size, so that larger foragers tended to travel faster and make fewer revisits than smaller foragers. Bumblebees show a continuous variation in body size that is primarily determined by the frequency of feeding so that larvae raised in the middle of the nest area (where workers are more active) tend to become the largest adults^53^. Size polymorphism is considered a main factor of caste determinism in bumblebees, such that only the largest individuals tend to undertake foraging the tasks^54^. Our novel results suggest that natural size variations also influence within caste behavioural variance among foragers. This observation is consistent with previous studies showing that the largest bumblebees make more foraging trips^55^, take less time^16^ and collect more nectar in natural conditions^16^. Large bumblebees also tend to learn faster in visual discrimination tasks^56^. These inter-individual behavioural and cognitive differences may be explained by differences in the sensory equipment of small and large bees. For instance, larger bees have bigger compound eyes and may thus be more accurate at finding small objects^57^. Size polymorphism in bumblebees is primarily determined by the frequency of feeding so that larvae raised in the middle of the nest area (where workers are more active) tend to become the largest adults^53^. Therefore it is very likely that the diversity of body sizes and their associated behavioural traits between and within castes of bumblebee colonies is a self-organised process, regulated by population densities and structural constraints within the nest at a given time during the colony cycle.

Our description of inter-individual variability in the spatial foraging behaviour of bumblebees is in line with recent observations that foragers of social bees show high variability to their contribution to the global colony foraging effort^55, 58^, suggesting that some behavioural traits may support higher foraging success. It has been suggested that behavioural diversity in a social group or population can be an advantageous trait at the collective level^7, 8^. Honey bee colonies showing higher genetic variability (and thus inter-individual behavioural variability) perform better in group tasks such as nest thermoregulation^59^. Colonies of *Thermothorax* ants showing high variability in the aggressiveness of workers are more productive^13^. In the social spider *Anelosimus studiosus*, mixed colonies composed of aggressive (asocial) and docile (social) individuals capture more prey than colonies with high proportion of only one type of individuals^60^. Accordingly, maintaining a diversity of behavioural profiles among foragers of a colony may allow the colony to locate and exploit a larger diversity of resources in fast changing environments^1, 24, 61, 62^. For instance, artificial bumblebee colonies containing individuals with different foraging profiles along a speed-accuracy trade-off have a more constant nectar collection rate than homogenous colonies^24^. Further investigation of the correlates of inter-individual behavioural and cognitive differences among members of a social group, such as bees, holds considerable promise for better assessing plastic collective responses and the adaptability of groups to stressful environmental conditions.

## Material and Methods

### Bees and flight room

We used two colonies of *Bombus terrestris* (Biobest, Westerlo, Belgium). Only one colony was tested at a time (colony 1: November-December 2015, colony 2: May-June 2016). We did not anticipate seasonal effects when working with commercially reared bumblebees in controlled laboratory conditions^27^. The colony was maintained in a two-chamber wooden nest box placed in an experimental flight room with white walls (length: 683 cm, width: 516 cm, height: 250 cm; Fig. 1). Controlled illumination was provided by 12 wide-spectrum light-emitting diode bulbs mimicking sunlight (15 W, 1250 lm, Ilight, Italy), with a 10 h: 14 h day: night photoregime (light on at 8:00 AM GMT + 1). Temperature was maintained at 20 °C. Bees were individually marked with numbered-colour tags (Opalith tags, Christian Graze KG, Germany) on their thoraces upon emergence from the pupae. The colony nest entrance was equipped with a transparent colourless Perspex tube with a series of shutters to control the traffic of foragers. Honey bee collected pollen was provided every two days directly into the colony nest box. Foragers collected sucrose solution (50% [w/w]) from artificial flowers in the flight room.

### Artificial flowers and landmarks

Each flower was made of a cylindrical plastic container (height: 7.5 cm, diameter: 6.2 cm) with a blue lid acting as a landing platform (Supplementary Fig. S1A). The platform was held 30 cm above ground by a clamp stand. We used two versions of this general flower design. “Pre-training” flowers provided bees with *ad libitum* reward through a cotton wick soaked in the flower’s container filled with sucrose solution (Supplementary Fig. S1B). “Training” flowers provided bees with a controlled volume of sucrose solution specific to each bee (range: 24–52 µL, N = 29 bees, see calculation of nectar crop capacity below). This volume was placed in the middle of the landing platform using an electronic micropipette (Handystep) (Supplementary Fig. S1C). We used nine three-dimensional landmarks made of cardboard and paper. Landmarks were uniquely defined by their shape and coloured patterns (Supplementary Fig. S2).

### Experimental procedure

Bees were allowed to forage collectively on a pre-training flower placed in the middle of the flight room (Fig. 1A). A regular forager that made at least five foraging bouts within one hour (flower visits followed by returns to the colony nest box) was selected for testing. The bee was first observed foraging on four training flowers arranged in a patch in the middle of the room (Fig. 1A). Each flower was refilled with 10 µL of sucrose solution by the experimenter immediately after being visited, until the bee returned to the nest. The average volume of sucrose solution collected by the bee over five foraging bouts was used to estimate its nectar crop capacity (range 48–208 µL, N = 29 bees)^31, 36–38^.

The bee was then tested for 20 consecutive foraging bouts in each of three experimental arrays on the same day (60 foraging bouts, ca. 6 h of observation per bee). Each array was characterised by a unique combination of four flower locations and four different landmarks (see details Fig. 1). All bees were tested in the same sequence (arrays 1, 2, 3). During the test, each flower provided a quarter of the bee’s crop capacity and was refilled by the experimenter between foraging bouts, so that the bee had to visit all flowers to fill its crop and return to the colony nest box. Because bumblebees drink sucrose rewards until their crop is full, any revisit to a flower within the same foraging bout was unrewarded^35–38, 63^. All flower visits, detailing the time when the bee landed on a flower and departed, and the time when the bee arrived and departed from the nest, were recorded using the software Ethom v.1.0^64^ (the complete flower visitation sequences are available in the Supplementary Dataset S1). Flowers were cleaned with ethanol solution (90% v/v) between changing arrays to preclude potential scent marks from influencing the bee’s flower choices in the new experimental array^65^. At the end of the test, the bee was freeze-killed and its body size (top of head to end of abdomen) measured with a digital calliper (±0.01 mm). A total of 29 bees were tested (14 workers from colony 1, 15 workers from colony 2). Bees from colony 1 were younger (age since emergence from the pupae (mean ± se); colony 1: 14.2 ± 8.66 days; colony 2: 24.5 ± 5.67 days, t-test: t = 6.61, df = 76, P < 0.001) and smaller (body length (mean ± se); colony 1: 13.41 ± 1.44 mm; colony 2: 16.13 ± 1.44 mm, t-test: t = 8.67, df = 82, P < 0.001) than bees from colony 2.

### Data analyses

#### Average foraging behaviour

All analyses were performed in R (version 3.2.3). We used regression models to describe changes in the average number of immediate revisits to flowers (two successive visits to the same flower), the average number of non-immediate revisits to flowers (two non-successive visits to the same flower), the average number of different flowers visited, and the average travel speed (flight duration divided by the Euclidian distance between all successively visited flowers), across the 20 foraging bouts of each bee in each experimental array. For each behavioural measure we ran both linear and logarithmic models and retained the model that had the highest R^2^ (Supplementary Table S1). We built a linear regression model using number of foraging bouts, identity of experimental arrays and the interaction between them as fixed effects. We examined the differences between experimental arrays using post-hoc Tukey tests (≪multcomp≫ R package^66^).

To assess the overall similarity between all flower visitation sequences of each bee in a given experimental array we used a determinism index (DET) derived from recurrence quantification analyses^44^. We compared the DETs calculated on the observed sequences to DETs calculated on 1000 randomly simulated sequences of 154 flowers - corresponding to the average number of flowers visits and nest returns over the 20 foraging bouts for all bees in each experimental array (mean ± se: 153.5 ± 33 visits, range = 107–286, N = 29 bees). The R code for generating random flower sequences is available in Supplementary Text S1. Observed and simulated DETs were compared using an analysis of variance (ANOVA) followed by a post-hoc Tukey test (≪multcomp≫ R package^66^). To compare the three observed DETs of the same bee (1 per experimental array), we applied a least-square means test (≪lsmeans≫ R package^67^) on a linear mixed effect model (LMM) including the experimental array as fixed effect and individual identity as random effect (≪nlme≫ R package^68^).

To examine whether some routes were more often used than others by the same bee, we focused on four-flower visitation sequences excluding revisits to flowers^31, 36–38^. We calculated the frequency of use of the primary route (highest proportion of foraging bouts in which the same four-flowers visitation sequence — excluding revisits to flowers — was used by a bee). Assuming that there are 24 (4! = 4 × 3 × 2 × 1) possible routes to visit four flowers once and return to the nest, we used a binomial test with a random probability of 0.042 (1/24) to use each route in a given foraging bout. Because each bee was tested for 20 foraging bouts in an experimental array, routes that were used at least four times by the same bee were used significantly more often than expected by chance (at the 5% level).

#### Intra- and inter-individual variability in foraging behaviour

We compared the foraging behaviour of individual bees using a principal component analysis (PCA). This PCA aimed to reduce our predictors (i.e. travel speed, number of different flowers visited, non-immediate revisits to flowers, immediate revisits to flowers, proportion of primary route usage, DET) to compound behavioural axes. We applied the Kaiser-Guttman criterion to select the number of principal components (PCs) to retain^69^. We then run the PCA function from the ≪psych≫ R package^70^ with only the retained PCs. We extracted the PC scores for each bee and used them as dependent variables in the subsequent analyses. To identify the effect of inter-individual (amount of variation among individuals around the average behaviour) and intra-individual (phenotypic plasticity of each individual across arrays) variability on the two PC components over the three experimental arrays of flowers, we ran mixed linear models (LMMs) with individual identity nested within colony identity as random effects. To do this, we ran both a random intercept (inter-individual variability) and slope (intra-individual variability) mixed effect model. We used individual age, body size and experimental array as fixed effects in order to evaluate their respective influence on both PCs. To assess inter-individual differences we tested for the significance of random intercept effects by applying a likelihood ratio test (LRT), comparing the LMM with individual identity nested within colony, the LMM with only colony as random effect and the linear model (LM) excluding both individual and colony identity. To quantify inter-individual variability, we calculated individual repeatability as the percentage of total variance explained by both colony origin and individual differences^71^. We also ran these two analyses on the slope models in order to assess the level of intra-individual variability over the three arrays.

## Electronic supplementary material

Supplementary Materials

Dataset S1

Dataset S2
