# Supplementary material for: Inter-individual variability in the foraging behaviour of traplining bumblebees
Source: Sci Rep. 2017 Jul 4;7:4561. doi: 10.1038/s41598-017-04919-8 (PMC5496863; doi:10.1038/s41598-017-04919-8)
Supplement: Supplementary file 1 — Supplementary Materials [file 41598_2017_4919_MOESM1_ESM.doc]

**SUPPLEMENTARY MATERIALS**

**Inter-individual variability in the foraging behaviour of traplining bumblebees**

Simon Klein^1,2^*, Cristian Pasquaretta^1^, Andrew B. Barron^2^, Jean-Marc Devaud^1^, Mathieu Lihoreau^1^

^1^ Research Center on Animal Cognition, Center for Integrative Biology, National Center for Scientific Research (CNRS), University of Toulouse (UPS), France

^2^ Department of Biological Sciences, Macquarie University, NSW, Australia

*Corresponding author: Simon Klein (simon.klein@univ-tlse3.fr)

Table S1: Choice of regression models. R^2^ were calculated for linear and logarithmic models for each behavioural measure in each experimental array. The model with the highest R^2^ (bold) was retained for the analyses.

| **Experimental array** | **1** | | **2** | | **3** | |
| --- | --- | --- | --- | --- | --- | --- |
| *Model type* | *linear* | *logarithmic* | *linear* | *logarithmic* | *linear* | *logarithmic* |
| Travel speed | 0.802 | **0.871** | 0.390 | **0.513** | 0.459 | **0.636** |
| Different flower locations visited | **0.773** | 0.740 | 0.215 | **0.289** | 0.419 | **0.547** |
| Immediate revisits | 0.514 | **0.829** | 0.297 | **0.555** | 0.268 | **0.594** |
| Non-immediate revisits | **0.394** | 0.388 | 0.291 | **0.512** | 0.017 | **0.079** |

Table S2: Differences in behavioural measures between experimental arrays. Post-hoc Tukey tests. Results in bold represent significant differences.

|  | β (SE) | t | P |
| --- | --- | --- | --- |
| *Travel speed* |  |  |  |
| **Array 1 *vs* array 2** | **0.15 (0.06)** | **2.60** | **0.032** |
| **Array 1 *vs* array 3** | **0.20 (0.06)** | **3.49** | **0.003** |
| Array 2 *vs* array 3 | 0.05 (0.06) | 0.89 | 0.65 |
| *Different flowers visited* |  |  |  |
| **Array 1 *vs* array 2** | **0.33 (0.06)** | **5.32** | **<0.001** |
| **Array 1 *vs* array 3** | **0.28 (0.06)** | **4.53** | **<0.001** |
| Array 2 *vs* array 3 | -0.05 (0.06) | -0.79 | 0.711 |
| *Immediate revisits* |  |  |  |
| Array 1 *vs* array 2 | -0.46 (0.23) | -2.01 | 0.124 |
| **Array 1 *vs* array 3** | **-0.80 (0.23)** | **-3.45** | **0.002** |
| Array 2 *vs* array 3 | -0.35 (0.23) | -1.53 | 0.285 |
| *Non-immediate revisits* |  |  |  |
| Array 1 *vs* array 2 | 0.77 (0.49) | 1.57 | 0.372 |
| **Array 1 *vs* array 3** | **-1.27 (0.49)** | **2.57** | **0.034** |
| **Array 2 *vs* array 3** | **-2.04 (0.49)** | **-4.14** | **<0.001** |

Table S3: Principal Components Analysis (PCA) loadings. For each individual in each experimental array, the average travel speed, number of immediate revisits to flowers, number of non-immediate revisits to flowers, number of different flowers visited per foraging bout were included in the PCA. The cumulated frequency of primary route usage and the determinism index (DET) of each bee in each array were also included. The correlation matrix of the six behavioural measures is showed in Supplementary Fig. S3.

|  | **Route fidelity (PC1)** | **Foraging performance (PC2)** |
| --- | --- | --- |
| Travel speed | -0.34 | -0.69 |
| Immediate revisits to flowers | -0.12 | 0.79 |
| Frequency of primary route usage | 0.81 | -0.09 |
| DET | 0.87 | 0.02 |
| Non-immediate revisits to flowers | -0.55 | 0.59 |
| Different flower locations visited | 0.36 | -0.49 |
| Proportion explained | 0.54 | 0.46 |
|  |  |  |

*Figure S1*


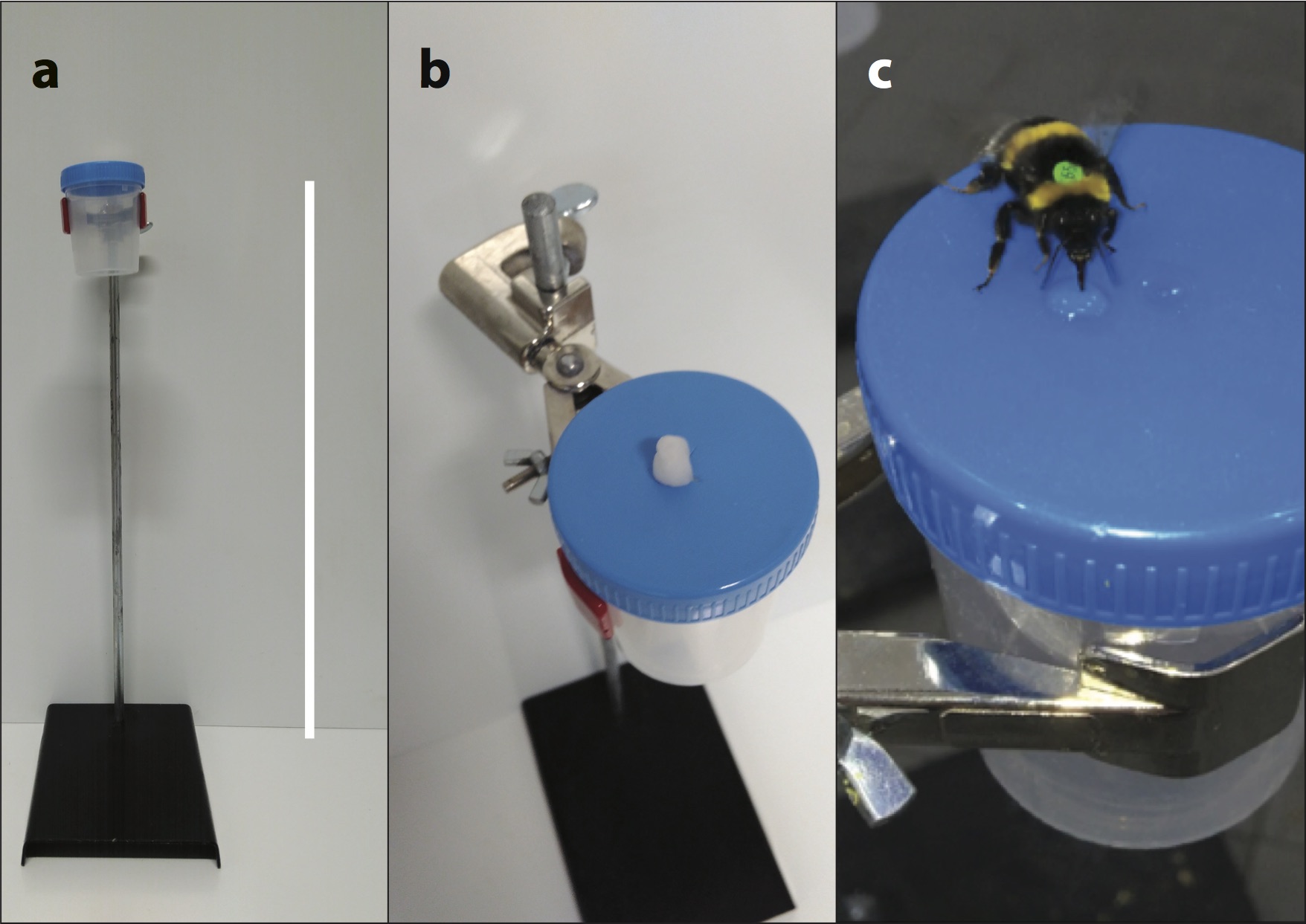


Figure S1: Photos of the artificial flowers. **a.** General flower design. The flower is made of a blue circular landing platform on top of a transparent, colourless, cylindrical reservoir of sucrose solution held by a stand clamp. White bar = 30 cm. **b**. Pre-training flower. Bees can drink *ad libitum* sucrose solution through the cotton wick connecting the landing platform to the sucrose reservoir. **c**. Training flower. A bee with a coloured numbered tag is drinking a controlled volume of sucrose solution placed in the middle of the landing platform. The bee cannot access the sucrose reservoir below. Pictures by S. Klein.

*Figure S2*


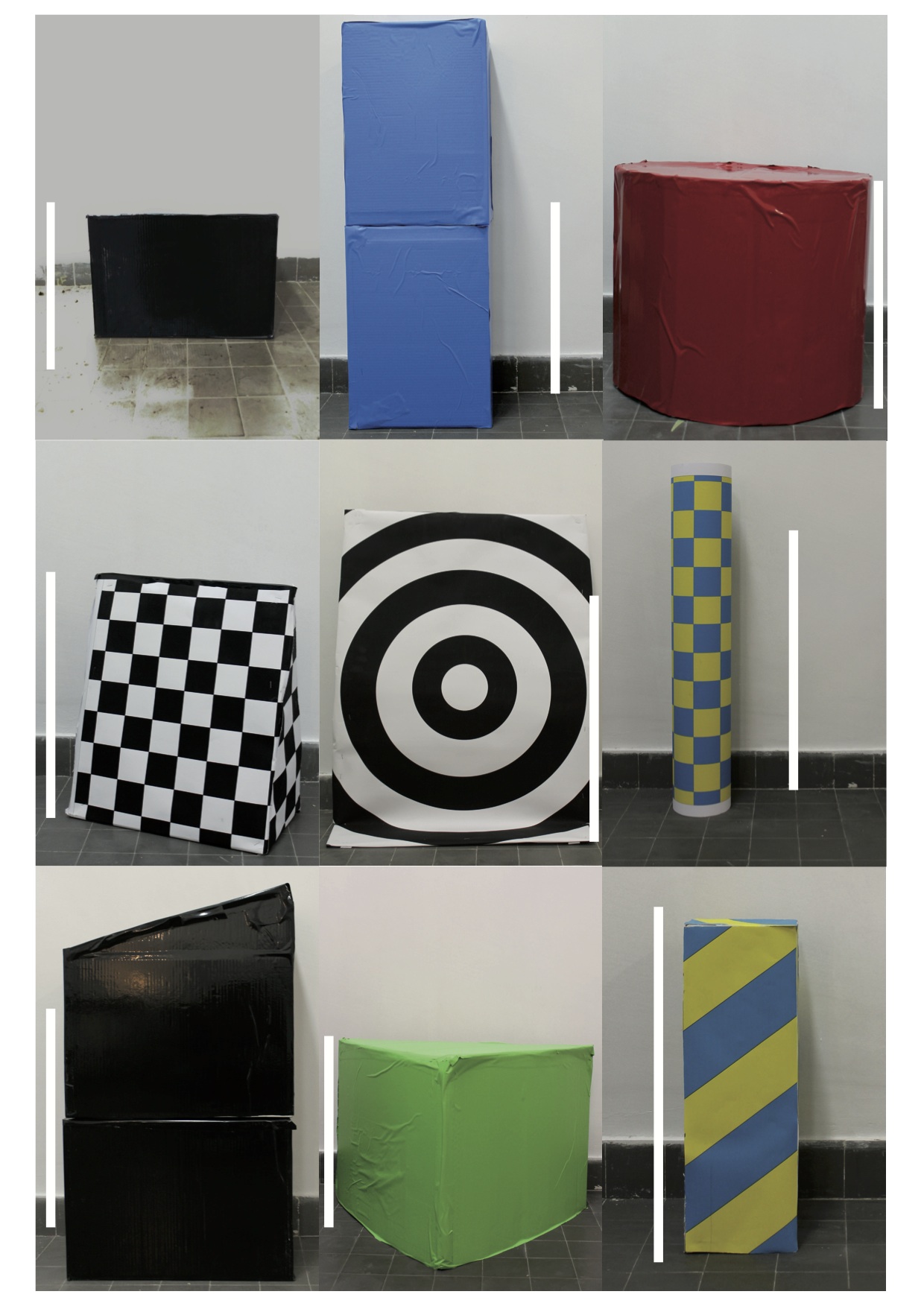


Figure S2: Photos of the three-dimensional landmarks. We used nine items made of cardboard and paper that could be used by bees as visual landmarks to assist their navigation. Each landmark was uniquely defined by its shape and colour pattern. White bar = 30 cm. The spatial arrangements of landmarks in the flight room are showed in Fig. 1. Pictures S. Klein.

*Figure S3*


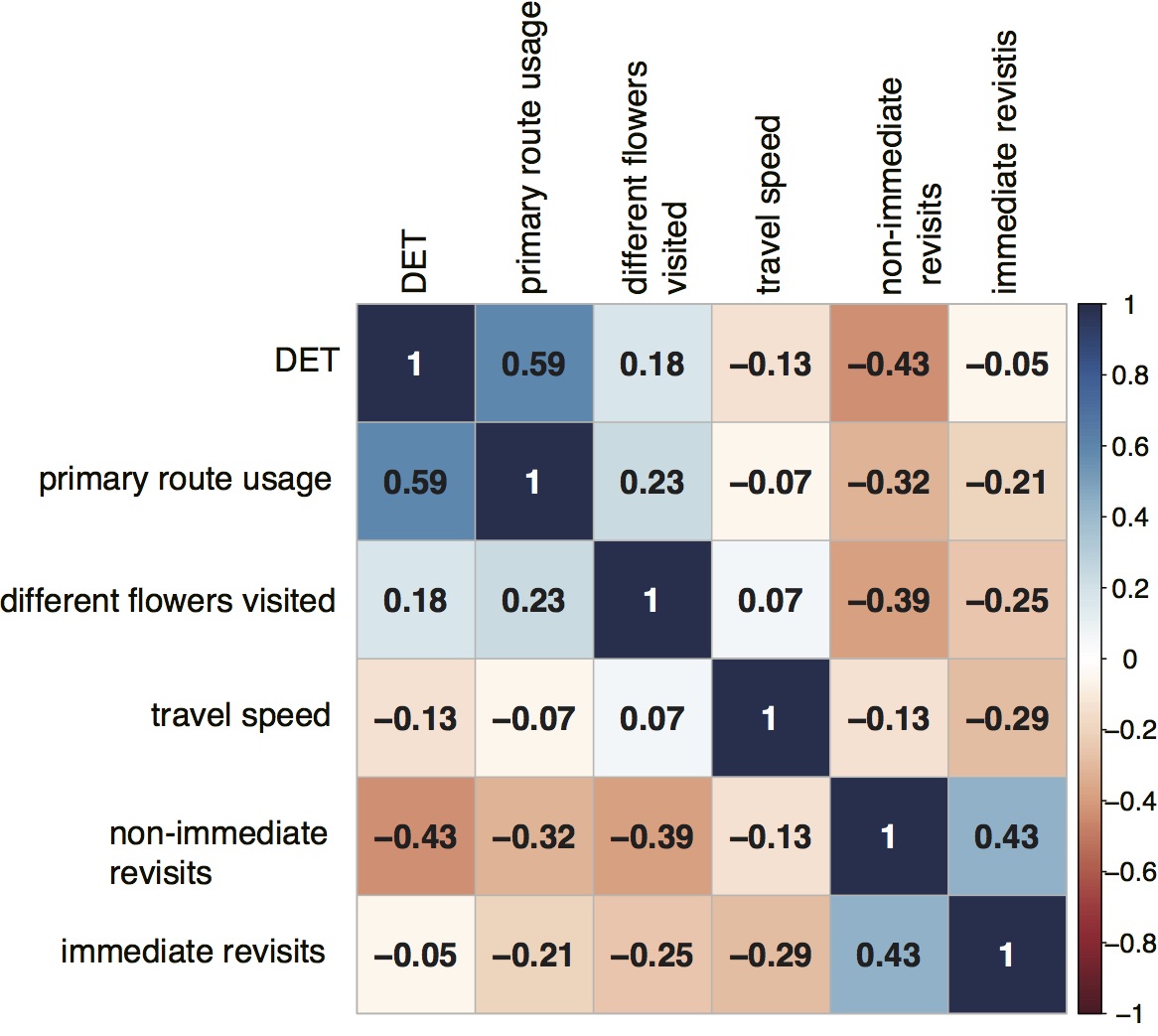


Figure S3: Correlation matrix of the six behavioural variables included in the principal component analysis. Travel speed per foraging bout (flight duration divided by the Euclidian distance between all successively visited flowers); number of different flowers visited per foraging bout; number of immediate revisits to flowers per foraging bout (when the bee visited the same flower twice in a row); number of non-immediate revisits per foraging bout (when the bee revisited a flower after having visited one or more different flowers); cumulative frequency of primary route usage per foraging bout; determinism index (DET, level of similarity between the 20 flower visitation sequences) for each experimental array.

*Figure S4*

Figure S4: Selection of the principal components (PCs) based on the Kaiser-Guttman criterion. Two PCs with an eigenvalue higher than the average (red line) were retained to construct the principal component analysis.

Dataset S1: Data file (csv.file) containing mean values for the number of different flowers visited (*nb_flower*), the number of immediate revisits (*imm_*revisit), the number of non-immediate revisits (*non_imm_*revisit), the travel speed (*speed,* in m/s), the proportion of primary route usage (*prop*) and the determinant (*DET)* for each individual (*bee_ID*) in the three experimental arrays of flower (*array*). SE are provided for each variable (*se_variablename*). This file also contains information about the colony origin (*colony*), age (*age*) and body size (*body*) of bees. NA = non-available value.

Dataset S2: Data file (csv.file) containing raw values for the number of different flowers visited (*nb_flower*), the number of immediate revisits (*imm_revisit*), the number of non-immediate revisits (*non_imm_revisit*), the travel speed (*speed,* in m/s), the proportion of primary route usage (*prop*) and the sequence of flower visits (*sqce_tot*) for each foraging bout (*bout*) of each individual (*bee_ID*) in the three experimental arrays of flowers (*array*). This file also contains information about colony origin (*colony*), age (*age*) and body size (*body*) of bees. NA = non-available value.

Text S1: R scripts used for generating random flower visits sequences and calculate random DET.

#=================================================================#

#-This R code creates a simulation of 1000 individuals visiting --#

#- flowers in random sequences and calculates their determinism index --#

#=================================================================#

# load the 2 functions developed by Ayers et al. 2015.

source(file = "~/Documents/bumble_expe/scripts/functions/determinism.R")

source(file = "~/Documents/bumble_expe/scripts/functions/removeperpdiag.R")

# generate 1000 artificial bees

det_rdm <- 0

for (j in 1:1000){

seq_rdm=0

for (i in 1:158) # 158 is the mean number of flowers visited by the 29 bumble bees in our dataset

{

TMP1=sample (1:4,1) # we arbitrarily assigned successions of 3 visited flowers. The 4th visit can be either nest return or another flower visit.

TMP2=sample (c(1:4),1)

TMP3=sample (c(1:4),1)

TMP4=sample (c(0:4),1)

seq_rdm=c(seq_rdm,c(TMP1,TMP2,TMP3,TMP4))

}

seq_rdm <- c(seq_rdm,0)

print(seq_rdm)

det_rdm <- rbind(det_rdm,determinism(seq_rdm,4))

#generate DET values for each of the 1000 artificial bees

}

det_rdm2 <- det_rdm[-1]

write.csv(det_rdm2,file="det_rdm.csv")
